# Inflammation: The Cause of All Diseases

**DOI:** 10.3390/cells13221906

**Published:** 2024-11-18

**Authors:** Vivek P. Chavda, Jack Feehan, Vasso Apostolopoulos

**Affiliations:** 1 Department of Pharmaceutics and Pharmaceutical Technology, L.M. College of Pharmacy, Ahmedabad 380009, Gujarat, India; vivek.chavda@lmcp.ac.in; 2 School of Health and Biomedical Sciences, RMIT University, Melbourne, VIC 3083, Australia; jack.feehan@rmit.edu.au

## 1. Introduction

**Figure 1:**
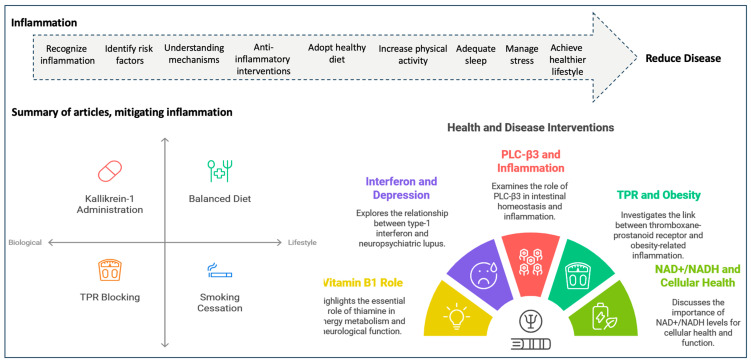
Process of managing chronic inflammation, and a summary of studies published in this Special Issue. Image was made using Biorender.com.

Inflammation is an essential biological process that serves as the body’s first line of defence against harmful stimuli, including pathogens, damaged cells, and irritants. While acute inflammation is crucial for healing and recovery, chronic inflammation can lead to a variety of diseases, including cancer, cardiovascular disorders, and autoimmune conditions [1,2,3]. The recent Special Issue, ‘Inflammation: The cause of all diseases 2.0’ [4], along with the participating journals—*Cells*, *Diseases*, *Healthcare*, *International Journal of Molecular Sciences*, and *Vaccines*—sheds light on the complex interplay between inflammation and disease mechanisms, providing valuable insights into potential therapeutic strategies targeting inflammatory pathways [5,6,7,8,9,10,11,12,13,14,15,16]. Inflammation is characterized by a series of physiological responses involving immune cells, blood vessels, and molecular and cellular mediators. When cells are injured due to external or internal stimuli, an inflammatory response is initiated involving the secretion of pro-inflammatory cytokines, chemokines, and other signalling molecules [17]. This response aims to eliminate the initial cause of injury, remove damaged cells, and initiate the repair process. However, when inflammation becomes dysregulated or persists beyond its intended duration, it can contribute to chronic tissue damage, chronic inflammation, and the development of chronic diseases such as type-2 diabetes (T2D) [5,18], cancer [19,20], chronic obstructive pulmonary disease (COPD) [21], and cardiovascular diseases (CVD) [22]. The articles featured in this Special Issue explore various aspects of inflammation, including its molecular mechanisms and implications for different pathological conditions (Figure 1). 

## 2. Chronic Inflammation Leading to Diseases

While chronic inflammation progresses quietly, it is a leading cause of many chronic diseases and poses a significant threat to individual health and longevity. Low-grade inflammation is increasingly recognized as a shared characteristic of metabolic, psychiatric, and neurodegenerative diseases [23,24]. Several factors promote low-level chronic inflammation, such as age, smoking, diet, sedentary lifestyle, obesity, hormones, stress, and irregular sleep patterns. Ageing correlates with an increase in inflammation due to the gradual decline in immune function with age (immune senescence), which leads to mitochondrial dysfunction, free radical accumulation, and increased visceral fat over time [25]. The chemicals in cigarette smoking stimulate the production of pro-inflammatory cytokines and reduce anti-inflammatory cytokines, contributing to chronic inflammation and leading to diseases such as cancer, chronic lung disorders, and vascular diseases [26]. Diet is well known to contribute to inflammation, especially highly processed foods and those rich in saturated fats, trans-fats, and refined sugar; the gut microbiota also play an important role in chronic inflammation [27]. In addition, sedentary patterns have been linked to low-grade inflammation and promote systemic chronic inflammation [28,29]. In a recent study, it was shown that regulatory T cells (Treg) suppress inflammation by enhancing exercise capacity and promoting muscle metabolic reprogramming by protecting mitochondria from interferon (IFN)-γ damage [30]. Physical activity has been shown to be beneficial in many disorders, including cancer, as well as in improving mental health and managing menopause symptoms [31,32]. Obesity leads to chronic low-grade inflammation, driven by pro-inflammatory immune cell activation and cytokine production, which contribute to insulin resistance and related diseases [33,34]. Both innate and adaptive immune responses, as well as adipokines, play significant roles in this process [35,36].

Twelve articles were published in this Special Issue, all of which discuss various molecular and cellular mechanisms through which inflammation drives chronic diseases. These are summarised as follows:

- Studies on chronic diseases: Excess type-1 interferon production is central to systemic lupus erythematosus, often resulting in neuropsychiatric lupus with depression. In this study, the administration of kallikrein-1 reduced type-1 interferon and improved depressive symptoms [7]. In another study, phospholipase C-β3 (PLC-β3), which plays a role in maintaining intestinal homeostasis, was shown to cause severe inflammation and lethality in PLC-β3 knockout mice [6]. This was observed after exposure to dextran sodium sulfate, which triggered inflammation in the small intestine due to disrupted Wnt/β-catenin signalling. Further, the reduced PLC-β3 levels in human ileal Crohn’s biopsies also supports its role in chronic inflammation and disease progression [6].
- Studies on diet: A balanced diet is crucial for preventing inflammation, as poor nutrition and unhealthy eating habits contribute to chronic inflammation [37]. Consuming a variety of nutrient-rich foods helps maintain overall health and supports the body’s ability to combat inflammatory processes, reducing the risk of chronic diseases associated with chronic inflammation. In the paper by Turrini et al., it was noted that vitamin B1 (thiamine) is essential for energy metabolism and neurological function, and thiamine deficiency can lead to Wernicke encephalopathy (WE). The authors reported a case of an 8-year-old girl with WE due to thiamine deficiency, presenting with ataxia, nystagmus, confusion, and other neurological symptoms, suggesting underlying chronic inflammation [16].
- Studies on obesity: In obesity-related diseases, there are elevated levels of several pro-inflammatory markers. As such, the link between thromboxane-prostanoid receptor (TPR) and obesity was evaluated, and it was noted that blocking TPR reduced lipopolysaccharide- and stearic acid-induced inflammation in human PBMCs; the activation of TPR enhanced these inflammatory effects [11]. In addition, in obesity, a high platelet–high-density lipoprotein ratio (PHR) is a potential marker of inflammation. Indeed, in 203 obese patients, higher PHR levels significantly correlated with the presence of T2D and glycaemic markers [5].
- Studies on smoking: Cigarette smoke from combustible cigarettes (CCs) contains harmful chemicals that impair immune cell function, exacerbating chronic inflammatory diseases. In contrast, heated tobacco products (HTPs) produce fewer harmful cytokines. The authors of this study found that both CCs and HTPs altered cytokine production in patients with ulcerative colitis, T2D, and COPD [9]. HTP, however, induced fewer pro-inflammatory cytokines but increased immunosuppressive IL-10, IL-35, and pro-fibrotic TGF-β [9]. In mice, exposure to cigarette smoke extracts, which induce inflammation in lung tissues, was mitigated by NADH supplementation [13]. NAD+/NADH levels are important for overall cellular health and function. This was shown by the improvements in lung antioxidant defences (superoxide dismutase, glutathione peroxidase, catalase, glutathione), reduced oxidative damage (malondialdehyde), and decreased pro-inflammatory markers (TNF-α, IL-17, IFN-γ, and myeloperoxidase activity) [13].
- Studies on immune/blood cells: In the paper by Tam et al. [15], genomic biomarkers were identified that are linked to high degranulation responses in primary human mast cells from 262 donors, suggesting that chronic inflammation, mediated by mast cell activation, can contribute to disease processes related to allergic responses. In addition, mild systemic inflammation increases erythrocyte fragility, potentially contributing to haemolysis. This was demonstrated in 9292 healthy participants where haemolysis was linked to high-sensitivity C-reactive proteins. A correlation with urinary neopterin/creatinine ratio and erythrocyte osmotic fragility was also noted in a mixed healthy population (n = 54) [14]. Both these findings link inflammation to impaired erythrocyte function. A review was published in this Special Issue describing the key players in rheumatoid arthritis pathogenesis. Immune cells, fibroblast-like synoviocytes, macrophages, monocytes, B cells, CD4+ T cells, Th1, Th17 cells, and pro-inflammatory mediators, CCR2, CX3CR1, RANKL, IL-1β, IL-6, and TNF-α, were described in the context of inflammatory responses associated with disease [12]. Another review described cellular senescence and mitochondrial dysfunction in CVD and provided advancements in therapies aimed at targeting mitochondrial dysfunction, such as energy starvation, oxidative stress, and mitophagy [8].
- Finally, a study by Popoca-Hernández et al. [10] evaluated non-surgical periodontal treatment in women with periodontitis and rheumatoid arthritis). The results showed significant reductions in periodontal indices, inflammatory biomarkers, and disease activity after non-surgical periodontal treatment. The findings highlight the critical role of oral health in managing chronic diseases like rheumatoid arthritis [10].

All the published papers highlight the role of chronic inflammation in driving disease, necessitating a deeper understanding of how such products affect immune responses, resulting in chronic diseases (Figure 1).

## 3. Therapeutic Applications

Low-grade chronic inflammation is key in the development of many diseases [38]. Unlike acute inflammation, which is a protective response to injury or infection, chronic inflammation persists over time and damages healthy cells and tissues, contributing to disease progression. Therapeutic interventions targeting chronic inflammation aim to modulate the immune system, reduce inflammatory markers, and restore normal tissue function. Common strategies include the use of nonsteroidal anti-inflammatory drugs (NSAIDs) [39], corticosteroids [40], biologic agents, and lifestyle modifications, all of which have shown promise in alleviating symptoms and preventing further tissue damage in chronic inflammatory diseases. Novel approaches to alleviating inflammation have been developed in recent years, with the aim of reversing or minimising progression to disease formation. One approach targeted the NLRP3 inflammasome, a key protein involved in inflammatory response, with small-molecule inhibitors [41]. NLRP3 inhibitors were able to reduce neuroinflammation and improve cognitive function in a mouse model of Alzheimer’s disease [42]. Metformin, an anti-diabetic drug, has been shown to have a dual purpose by downregulating the expression of pro-inflammatory markers (IL-1β, IL-6, TNF-α, COX-2, and TNF receptors) in cancer [43]. In addition, metformin reduces inflammation caused by air pollution in thrombosis [44], Parkinson’s disease [45], and lung injury [46]. Monoclonal antibodies such as infliximab, etanercept, adalimumab, golimumab, and certolizumab, which target pro-inflammatory cytokines such as TNF-α in autoimmune/inflammatory diseases like lupus, rheumatoid arthritis, ulcerative colitis, ankylosing spondylitis, and uveitis, have increasingly become popular due to their ability to improve disease outcomes by reducing systemic inflammation [47]. Targeting the JAK-STAT pathway has been investigated to manage chronic inflammation [48]. Furthermore, bio-active compounds and dietary polyphenols have been shown to reduce inflammation, and could therefore be used as a complementary therapy for inflammation-related diseases [49]. The gut microbiota have been linked to inflammation and interventions are being developed to modulate the gut microbiome, such as probiotics or prebiotics, which could potentially reduce reliance on anti-inflammatory drugs, for example, in asthma treatment [50]. The therapeutic landscape for inflammation-driven diseases is advancing rapidly. New biologics targeting specific cytokines, inflammasomes, and inflammatory signalling pathways, such as JAK-STAT, bio-active compounds, and microbiome modulation, highlight the growing potential of precision medicine. These innovations are key to reducing the burden of chronic inflammatory conditions and improving patient outcomes.

## 4. Conclusions

While inflammation is crucial for the healing process, chronic inflammation can lead to significant health problems. Chronic inflammation is linked to numerous diseases (‘all diseases’), ranging from CVD, T2D, metabolic diseases, cancer, autoimmunity, gastrointestinal disorders, respiratory diseases, neurodegenerative diseases, reproductive system disorders, allergies, skin disorders, and joint problems to headaches, food sensitivities, hormonal imbalances, and sleep disorders. The articles featured in this Special Issue provide valuable insights into the intricate relationship between inflammation and disease mechanisms, emphasizing how chronic inflammation underlies certain pathologies. Advancing the understanding of how inflammation drives various pathological conditions could pave the way for innovative therapeutic strategies, preventing the detrimental effects of chronic inflammation and ultimately improving health outcomes and quality of life. Addressing the core causes of inflammation may be crucial in preventing and treating many chronic diseases in the future.
